# Do Food Intake and Food Cravings Change during the Menstrual Cycle of Young Women?

**DOI:** 10.1055/s-0038-1675831

**Published:** 2018-11

**Authors:** Luciana Bronzi de Souza, Karine Anusca Martins, Mariana Morais Cordeiro, Ymárdila de Souza Rodrigues, Bruna Paola Murino Rafacho, Rafael Aiello Bomfim

**Affiliations:** 1 Faculty of Nutrition, Universidade Federal de Goiás, Goiânia, GO, Brazil; 2 Faculty of Pharmaceutical Sciences, Foods and Nutrition, Universidade Federal de Mato Grosso do Sul, Campo Grande, MS, Brazil; 3 Faculty of Dentistry, Universidade Federal de Mato Grosso do Sul, Campo Grande, MS, Brazil

**Keywords:** menstrual cycle, food intake, nutrition assessment, feeding behavior, luteal phase, follicular phase, ciclo menstrual, consumo alimentar, avaliação nutricional, comportamento alimentar, fase lútea, fase folicular

## Abstract

**Objective** The aim of the present study was to assess the anthropometric measures, food intake and food cravings during the menstrual cycle of undergraduate students of the faculty of nutrition.

**Methods** A cross-sectional study was performed with 27 students from a public university in the state of Mato Grosso do Sul, Brazil, who had their food intake evaluated through a 24-hour food recall, their nutritional status evaluated based on anthropometric measures, and food cravings evaluated using the Food Desire Questionnaire. Data were collected during an evaluation in the follicular phase (between the 5^th^ and the 9^th^ day of the menstrual cycle) and another in the luteal phase (LP) (between the 20^th^ and the 25^th^ day of the menstrual cycle). For food intake variables, the analysis of variance (ANOVA) test was used, followed by the Tukey test. The Mann-Whitney test was used for the analysis of food cravings, considering a significance level of 5% (*p* < 0.05).

**Results** The desire for foods rich in sugar, salt, and fat, such as chocolate, pastries, snacks and desserts were higher (*p* < 0.05) during the premenstrual period, although it did not reflect neither a higher energy intake nor an alteration in the distribution of macronutrients. A higher intake of carbohydrates, proteins, fibers, and calcium was observed during the LP; however, without statistical difference between the groups. There were no differences either in the intake of any food group or in the anthropometric measurements (*p* > 0.05).

**Conclusion** Food cravings of nutrition students differed between the phases of the menstrual cycle; however, with no difference in food intake and in anthropometric measures.

## Abstract (pt)

**Resumo:**

**Objetivo** Verificar alterações de medidas corporais, consumo e desejos alimentares durante o ciclo menstrual de acadêmicas de nutrição.

**Métodos** Estudo transversal com 27 estudantes de uma universidade pública do Mato Grosso do Sul, as quais tiveram seu consumo alimentar avaliado por meio de recordatório alimentar de 24 horas, estado nutricional avaliado com base em medidas antropométricas, e desejos alimentares avaliados utilizando-se o Questionário de Desejo Alimentar. Os dados foram coletados durante uma avaliação na fase folicular (entre o 5° e o 9° dia do ciclo menstrual) e outra na fase lútea (entre o 20° e o 25° dia do ciclo menstrual). Para as variáveis de consumo alimentar, utilizou-se o teste análise de variância (ANOVA, na sigla em inglês), seguido pelo teste de Tukey. Já para a análise dos desejos alimentares, utilizou-se o teste de Mann-Whitney. Foi considerado o nível de significância de 5% (*p* < 0,05).

**Resultados** Os desejos por alimentos ricos em açúcar, sal e gordura, como chocolates, produtos de pastelaria, lanches e sobremesas foram maiores (*p* < 0,05) no momento pré-menstrual, apesar de não refletirem em maior consumo energético e tampouco em alteração na distribuição de macronutrientes. Observou-se maior consumo de carboidratos, proteínas, fibras e cálcio na fase lútea; no entanto, sem diferença estatística entre os grupos. Não foram encontradas diferenças no consumo de nenhum grupo alimentar, tampouco nas medidas antropométricas (*p* > 0,05).

**Conclusão** Os desejos alimentares das acadêmicas de nutrição diferiram entre as fases; no entanto, sem diferença no consumo alimentar e nas medidas corporais.

## Introduction

Thousands of women of childbearing age experience some degree of premenstrual syndrome (PMS), which includes emotional, physical, cognitive and behavioral symptoms related to the menstrual cycle, such as irritability, depressive mood, changes in appetite, pain, and anxiety. These symptoms are recurrent during the luteal phase (LP) and usually remit within a few days after the onset of menstruation.^1^ Nutrition, stress, and emotion are environmental factors that can interfere with the menstrual cycle.^2^

According to the hormonal fluctuations, the menstrual cycle is divided into phases. In a simplified division, there are two phases, the follicular phase (FP) and the LP. The FP begins in the 1^st^ day of menstrual bleeding (1^st^ day of the cycle) and ends with the ovulation. The FP is characterized by increased secretion of estrogen, of follicle-stimulating hormone (FSH), and of luteinizing hormone (LH) just before ovulation. The LP follows the ovulation and is characterized by rising progesterone and estrogen levels. It is the only time during the cycle in which progesterone is unopposed by estrogen.^3^ The normal alteration in estrogen and progesterone hormone levels in the menstrual cycle appear to act on the serotonergic function, leading to manifestations of the symptoms of the syndrome.^1^

According to Reid (2017),^4^ during the reproductive years, up to between 80 and 90% of the women who menstruate feel some change during the premenstrual period, such as breast pain, bloating, acne and constipation. The incidence of severe symptoms ranges between 3 and 5% of the women of childbearing age, who are severely incapable of carrying out their lives during this phase of the cycle. Severe symptoms are also responsible for causing changes in their family, social and professional life for approximately two weeks at the beginning of each month. Epidemiological surveys have shown that between 75 and 80% of the women have symptoms in the premenstrual period.^5^ Among the main symptoms described in the period of PMS, there are changes in mood, depression, low self-esteem, irritation, anxiety, nervousness, aggression, sensitivity to emotions, impulsive behavior, body aches, fatigue, insomnia, changes in appetite, and binge eating of sweet or salty foods as the main symptoms described during the PMS period.^1^

Many women change their eating habits during the phases of the menstrual cycle, especially in relation to the consumption of chocolates, sweets in general, and salty foods.^6^ The increased intake of carbohydrates in the LP can be justified by the reduction of serotonin mediators in this period. The increased production of serotonin relieves symptoms, so craving for sweet foods like chocolate would be an unconscious way of improving such symptoms, since by increasing serotonin levels, a balance would be achieved as a form of relief,^7^ so eating usually reduces irritability or promotes positive affect.^8^

Estrogens and progestogens have been linked to disordered eating during the LP. Klump et al (2013)^9^ discovered that in a community sample, there were day-to-day associations between ratings of emotional eating and estradiol and progesterone levels.

Although data from the literature^6^ ^10^ demonstrate the existence of changes in the eating behavior of women during the menstrual cycle, there are few studies that address these changes in Brazilian women. Therefore, the present study aims to evaluate the alteration of dietary intake, food cravings, and nutritional status in the stages of the menstrual cycle of nutrition students from a public university in Campo Grande, state of Mato Grosso do Sul, Brazil.

## Methods

This cross-sectional study was conducted between March and June of 2017 in a public university, with students enrolled in the nutrition undergraduate course.

The participants were regularly menstruating (25–35 days, as self-reported) premenopausal women recruited from the undergraduate course in nutrition. All the female students regularly enrolled were invited to participate through electronic mail and visits to classrooms. Other inclusion criteria were being in childbearing age and > 18 years old. Students were excluded if they were pregnant or lactating, taking oral contraceptives, hormone supplements, or any weight- or hydration status-modifying drugs (cortisone, antidepressants and others), or if they were on any calorie-restricted diet.

When an interested student accepted to attend the project and followed the criteria for inclusion and exclusion, the investigator scheduled an initial assessment. During that assessment, the women received instructions regarding the study procedures, signed a written consent form and completed questionnaires about demographic information such as age, marital status, and in which period they were enrolled in the course. Information regarding food intake, food cravings, and the anthropometric evaluation were scheduled according to the onset of menstruation of each participant: one in the FP (from the 5^th^ to the 9^th^ day of the menstrual cycle) and another in the LP (from the 20^th^ to the 25^th^ day of the menstrual cycle). They were classified in phases according to the onset of menstruation.

To measure the dietary intake, participants completed two 24-hour recalls (R24H): one in the LP and another in the FP. DietPro Clinico software version 5.8 (DietPro, Viçosa, MG, Brazil) was used to analyze the dietary intake and to obtain the caloric value of the diet, as well as the percentages of energy derived from carbohydrates, proteins and lipids. Food was classified according to the food group, considering the Food Guide for the Brazilian Population.^11^

Food cravings were also assessed in both phases by the Food Desire Questionnaire.^12^ The questionnaire was minimally adapted with local foods and comprised a list of 38 foods and beverages, as in the original version. The participants were asked to rate how much they would like to eat each of the items in the questionnaire, on a 5-point scale, ranging from 0 (no desire) to 4 (very high desire).

For the anthropometric evaluation, body weight (kg), height (m) and waist circumference (WC) (cm) were measured in both phases of the cycle. All of the measurements were performed according to the standards of the Food and Nutrition Surveillance System (SISVAN, in the Portuguese acronym).^13^ The data collection procedures were performed at the nutrition evaluation laboratory of the faculty of pharmaceutical sciences, food and nutrition of the Universidade Federal do Mato Grosso do Sul.

The results were presented through descriptive statistics, as arithmetic mean and standard deviation (SD). For food consumption variables, as the data presented a normal curve (Kolmogorov-Smirnov), a parametric statistic was done, using the analysis of variance (ANOVA) test, followed by the Tukey test. For the food cravings analysis, non-parametric analyses were performed (non-normal curve), using the Mann-Whitney test. In all tests, a significance level of 5% or corresponding *p*-value and 95% confidence interval (CI) were used. Stata Statistical Software release 14 (StataCorp, College Station, TX, USA) was used to perform the statistical analyses.

The study started after receiving the approval of the study protocol by the Research Ethics Committee of the Universidade Federal do Mato Grosso do Sul, under the protocol no. 1.936.178/2017. All of the volunteers freely consented to participate by signing an informed consent form after the clarification of the purpose of the study and authorized the use of the data under the guarantee of anonymity and confidentiality, following the Resolution 466 of 12/12/2012.

## Results

Considering the 116 female students enrolled in the nutrition undergraduate course who were invited by e-mail to participate, only 27 were eligible and concluded the full protocol of evaluation. Subjects withdrew from the study due to non-compliance with the study protocol, personal reasons, and time conflict. The mean age of the participants was 21.85 years old (standard deviation [SD] = 0.54). The majority of the students reported being single (81.48%).

Among the participants, 33.33% (*n* = 9) were in the first year of the course, 29.63% (*n* = 8) were in the third year, 25.93% (*n* = 7) were in the fourth year, and 11% (*n* = 3) were in the fifth year. There were no participants in the second year of the course. The minimum duration of the course is 5 years.

### Anthropometric Data

The anthropometric data of the 27 participants are presented in Table 1. The mean height of the participants was 1.61 m (SD = 0.13).

**Table 1:** Anthropometric data of nutrition undergraduate students (*n* = 27) during the luteal and follicular phases of the menstrual cycle

Variable	Mean (SD) LP	Mean (SD) FP	*p-value*
Weight (kg)	60.91(2,68)	60.67 (2,61)	0.4781
BMI (kg/m^2^)	23.47 (0,89)	23.38 (0,86)	0.3462
WC (cm)	75.89 (1,94)	75.26 (1,95)	0.1183

Abbreviation: BMI, body mass index; FP, follicular phase; LP, luteal phase; WC, waist circumference.

### Effect of the Menstrual Cycle on Food Intake

The average intake of calories, macronutrients, fibers, calcium, and iron are presented in Table 2. A higher intake of calories (kcal/day), proteins (g/day), carbohydrates (g/day), fibers (g/day), and calcium (mg/day) during the LP can be observed. However, this difference was not significant for any of the variables analyzed during the phases of the menstrual cycle.

**Table 2:** Energy, macronutrients, fibers, calcium and iron intake during the luteal and follicular phases of the menstrual cycle of nutrition undergraduate students (*n* = 27)

Variable	Mean (SD) LP	Mean (SD) FP	*p-value*
Kcal/day	1,737.96 (413.66)	1,693.60 (436.51)	0.3832
Carbohydrates (g)/day	226.46 (67.27)	219.23 (69.51)	0.3881
Protein (g)/day	70.22 (24.73)	67.14 (20.70)	0.4973
Lipids (g)/day	61.27 (18.73)	62.37 (19.69)	0.2112
Fiber (g)/day	16.72 (9.51)	14.93 (9.10)	0.7056
Calcium (mg)/day	537.75 (292.14)	514.34 (249.74)	0.3164
Iron (mg)/day	10.44 (3.94)	10.52 (3.46)	0.0793

Abbreviations: FP, follicular phase; LP, luteal phase; SD, standard deviation.

**Fig. 1:**
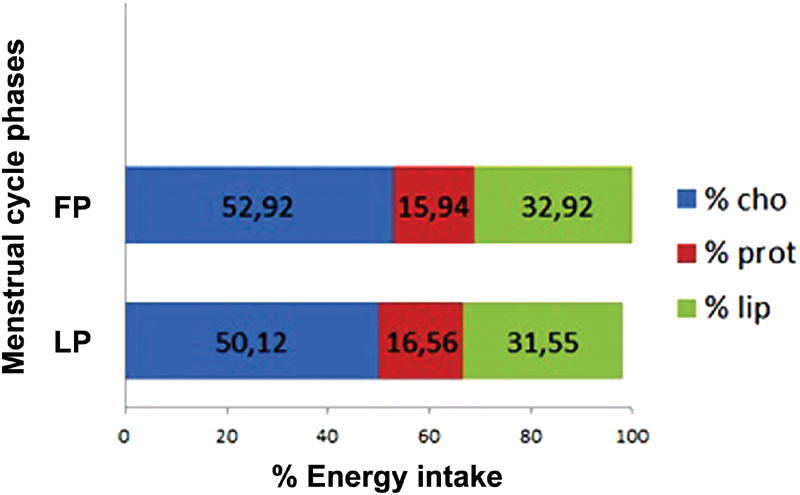
Mean percentage of energy from carbohydrates (% cho), protein (% prot) and lipids (% lip) during the phases of the menstrual cycle of nutrition students (*n* = 27). Abbreviations: cho, carbohydrates; FP, follicular phase; lip, lipids; LP, luteal phase; prot, protein.

Considering the percentage of energy from the macronutrients, the results can be observed in Fig. 1. There was no statistically significant difference between the percentages of energy from the macronutrients during the menstrual cycle phases of the nutrition students.

**Fig. 2:**
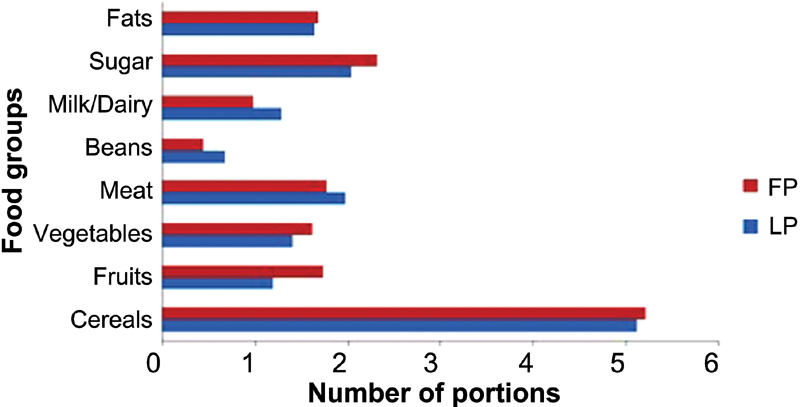
Comparison between the number of portions consumed in the luteal and follicular phases of nutrition students (*n* = 27). Abbreviations: FP, follicular phase; LP, luteal phase.

In relation to the portions of food consumed, classified according to the Food Guide for the Brazilian Population, Fig. 2 shows the comparison between the number of portions consumed in the LP and in the FP. No significant difference was found in the consumption of any of the food groups during the phases of the menstrual cycle (*p* > 0.05).

### Food Desire Questionnaire

**Fig. 3:**
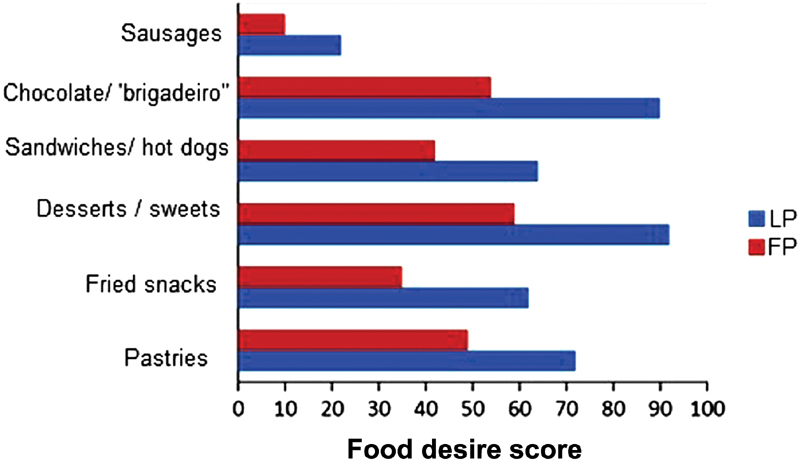
Foods that have changed the desire to be consumed during the phases of the menstrual cycle in nutrition students. *“brigadeiro”: a typical Brazilian dessert, made of chocolate and condensed milk. Abbreviations: FP, follicular phase; LP, luteal phase.

The findings referring to the food cravings during the phases of the menstrual cycle are represented in Fig. 3. The foods presented in Fig. 3 are those with a statistically significant change in the desire to be eaten. The food craving for pastries (*p* = 0.002), fried snacks (*p* = 0.01), desserts and sweets (*p* = 0.0002), sandwiches and hot dogs (*p* = 0.001), chocolate and “brigadeiro” (a typical Brazilian dessert, made of chocolate and condensed milk) (*p* = 0.0001), and sausages (*p* = 0.04) was higher during the LP when compared with the FP of the menstrual cycle. A tendency for higher intake of ice cream (*p* = 0.06) and of foods like French bread, soda, and alcoholic beverages (*p* = 0.07) was observed.

## Discussion

According to the classification of body mass index (BMI), the majority of the participants was classified as eutrophic (59.26%), 18.52% were classified as overweight, 14.81% were classified with obesity, and 7.41% with leanness, according to the World Health Organization (WHO).^14^ The results of the present study showed that there was no statistically significant difference between food intake and the anthropometric evaluation during the LP and the FP of the menstrual cycle of the nutrition students. However, the food cravings differed significantly between the two phases. Measurements of weight and of BMI were not altered during the menstrual cycle phases, differently from those described in the literature.^15^

Weight variation during the menstrual cycle is commonly reported, and one of the causes for such an outcome would be an increase in energy intake due to the increased appetite caused by hormonal oscillation.^16^ ^17^ In the present study, there was no change in energy intake, which may explain the absence of alterations in the anthropometric measures analyzed.

The food intake did not present change between the LP and the FP. Although the intake of calories (kcal/day), proteins (g/day), carbohydrates (g/day), fibers (g/day), and calcium (mg/day) during the LF was higher, it was not statistically different. There was also no significant difference in the intake of any of the food groups during the phases of the menstrual cycle (*p* > 0.05).

In the literature, there is no consensus on the impact of the menstrual cycle on food intake. The general consensus is an increase in energy intake during the premenstrual period compared with the postmenstrual period, although reports on macronutrients are less consistent. In studies with similar methodologies, authors^15^ ^18^ ^19^ reported a higher caloric intake in the LP, although these increases have not always been statistically significant. There are also some studies that found no differences in caloric intake or even an alteration in the intake of food groups.^20^ ^21^ In Brazil, there is a lack of information on these modifications during the menstrual cycle of healthy women.

Data from the literature describe results similar to those found in the present study,^15^ ^20^ in which there was no statistically significant difference in the caloric intake before and after the menstrual period. The mean difference observed in the present study is 44 kcal between the phases.

In the present study, there is a difference of 7 g of carbohydrate intake between the LP and the FP, which is very close to that found in a study that evaluated Arab women.^15^ According to the author of this study, a difference of 9g of carbohydrate intake was observed between the phases of menstrual cicle was observed, despite different eating habits. Moreover, this increase in carbohydrate intake would subjectively justify the greater sense of the need to consume their food source, like the foods identify as the greater food craving, like chocolate, desserts, pastries among others. Regarding the type of carbohydrate consumed in the premenstrual period, a previous study reported that sources of simple carbohydrates present a higher intake than the sources of complex carbohydrates.^20^ ^21^

One of the hypotheses to be considered for the increase in carbohydrate intake in this period would be the relationship between simple carbohydrates (high glycemic index) and a higher production of cerebral serotonin,^22^ thus reducing the negative mood effects.^23^ The second hypothesis to be considered is the relationship between carbohydrate intake and the menstrual cycle, since there is a variation in the concentration of steroids, and it is observed that estrogen suppression may influence the intake of carbohydrates.^15^

However, in a study comparing food intake, nutrients, and serum levels of estrogen, progesterone and leptin during the phases of the menstrual cycle in 39 Thai women aged between 20 and 40 years old, the authors reported that, although consuming more total calories (+ 160 kcal/day, *p* < 0.05) and more grams of proteins (+ 6–8 g/day, *p* = 0.01) during the LP when compared with the FP, no correlations were observed between the serum levels of sex hormones, serum leptin levels, food intake, or body weight.^24^

Regarding the food groups, there was no change in the intake of any of the analyzed groups. In a study performed with 45 university students from the faculty of nutrition of the Universidade Federal Fluminense,^21^ it was observed that there was no statistically significant difference in the intake of the food groups in the menstrual cycle, similar to the results described here, except for the group considered complementary and beverages (with high fat, sugar and salt content), whose intake increased (*p* = 0.04) during the LP.

Despite the fact that no change in the intake of energy, of nutrients and of food groups was observed, food cravings had a significant alteration. When the pre-and postmenstrual food craving was analyzed, there was a significant variation in the desire to eat caloric foods rich in simple carbohydrates, salt, and sugar, such as pastries, snacks, sausages, chocolate, sweets, and desserts in general. Other studies that evaluated the impact of the menstrual cycle on food cravings found results similar to those of the present study.^6^ ^21^

The mechanism underlying the selective effect of these symptoms of the LP on the desire to eat highly sweet foods is unclear. According to Gibson (2006),^8^ sweet foods may improve emotion and mitigate the effects of stress through brain opioidergic and dopaminergic neurotransmission. Repeated short-term positive experiences after the ingestion of sweet foods might create positive emotional expectations for the ingestion of sweets. If so, more depressed women might engage in more emotional eating to alleviate their negative emotion.^25^

Impulsivity and irritability, symptoms that are commonly described as exacerbated in the LP, are associated with the desire to eat highly sweet foods, because of its emotional responses, which increase the risk of obesity.^26^ On the other hand, during the FP, estrogens could progressively decrease eating and the preference for sweet foods.^27^

Qualitative results^28^ showed that there is preference for the intake of sweets, carbohydrates, and foods with high fat contents. In the literature,^10^ ^20^ some researchers found that the desire for food, especially those containing fat, were significantly higher in the LP when compared with the FP. There was also a higher intake of foods such as sweets, sugars, and fats in the LP.^21^ Corroborating with the current study in relation to food cravings, another author^29^ applied a questionnaire to 52 women and found that 49% of them had a higher desire to eat sweet foods and that 37% reported a higher than usual food intake.

The present study backs up the findings of a study^21^ that also evaluated nutrition academics and found similar results of both intake and anthropometry. However, these results differ from what has been described in the literature when randomly selected women in the community were evaluated.^15^ ^20^ ^23^ ^29^

Despite the desire for high-calorie, high-fat, and high-sugar foods during the LP, this desire did not effectively change the intake due to the food control that may exist in the study group, since the participants in the study are academics of the nutrition course and, therefore, know the importance and principles of healthy eating.

However, these marked changes in appetite during the LP is described in the literature, as well as specific food cravings.^30^ Therefore, population studies need to be conducted to better clarify the mechanisms involved in this selected food preference, not only for sweet foods in general, but also for high-fat and high-calorie foods, thus providing improvements in health care and in the quality of life of this group.

Nevertheless, it is important for health professionals, especially nutritionists and gynecologists, to know the possible oscillations that may occur in the premenstrual period and to consider them for research on food intake and food craving, as well as to provide differentiated care at this stage.

Several limitations of the present study should be noticed. First, the number of participants is limited. Despite the number of female students enrolled in the course (*n* = ∼ 116), only 27 completed the full study protocol, that is, performed the evaluation at both moments: during the LP and the FP of the menstrual cycle. The need to perform two evaluations at different times, as well as the exclusion criteria, made it difficult to perform the follow-up of the participants of the present study. Second, the absence of a test to confirm in which phase of the cycle the participants were in, which was classified according to the onset of the menstruation. Third, the Food Desire Questionnaire was minimally adapted from the original version with local foods, but this adaptation had not been validated in a previous study.

## Conclusion

The menstrual cycle presents itself as a unique model for regulating food intake and craving. The findings of the present study suggest that, during the menstrual cycle, the participants presented alterations in food craving, with a higher desire for caloric foods, as well as for foods rich in fats, sugars and salt during the LP compared with the FP, thus identifying PMS as a possible determinant of dietary desires in women of childbearing age. However, despite the changes in food craving, the total intake of calories and also of macro and micronutrients did not fluctuate across the menstrual cycle. There was also no fluctuation in body weight, BMI, or WC. This fact may be a reflection of food control.
